# Non-Inherited Maternal Antigens Identify Acceptable HLA Mismatches: A New Policy for the Hellenic Cord Blood Bank

**DOI:** 10.3390/bioengineering5040077

**Published:** 2018-09-21

**Authors:** Effrosyni Panagouli, Amalia Dinou, Panagiotis Mallis, Efstathios Michalopoulos, Andreas Papassavas, Maria Spyropoulou-Vlachou, John Meletis, Maria Angelopoulou, Kostas Konstantopoulos, Theodoros Vassilakopoulos, Catherine Stavropoulos-Giokas

**Affiliations:** 1 Hellenic Cord Blood Bank, Biomedical Research Foundation Academy of Athens, 4 Soranou Ephessiou Street, 11527 Athens, Greece; epanagouli@bioacademy.gr (E.P.); adinou@bioacademy.gr (A.D.); pmallis@bioacademy.gr (P.M.); andreas.papasavvas@yahoo.com (A.P.); cstavrop@bioacademy.gr (C.S.-G.); 2 Immunology Division, General Hospital of Athens “Alexandra”, Lourou Street, 11528 Athens, Greece; marilynspy@yahoo.gr; 3 Department of Hematology and Bone Marrow Transplantation, School of Medicine, National and Kapodistrian University of Athens, 17, Agiou Thoma Street, 11527 Athens, Greece; imeletis@med.uoa.gr (J.M.); mkangelop@gmail.com (M.A.); kkonstan@med.uoa.gr (K.K.); theopvass@hotmail.com (T.V.)

**Keywords:** cord blood, NIMA, Hellenic Cord Blood Bank, CBU transplantation

## Abstract

Background: During pregnancy, the maternal-fetal contact may lead to the development of tolerance against the maternal human leukocyte antigen (HLA) that is not inherited by the fetus. These non-inherited maternal antigens (NIMAs) define acceptable HLA mismatches; therefore, the number of HLA phenotypes that are suitable matches for patients who need a hematopoietic stem cell transplant could be increased. Cord blood unit (CBU) transplantations to patients mismatched for a HLA loci, but similar to the ΝΙΜAs of the CBU, have a prognosis similar to 6/6-matched ones. Methods: The Hellenic Cord Blood Bank (HCBB) identified the maternal HLA of 380 cord blood donors, specifying the NIMA haplotypes of the related cryostored CBUs. Results: The HCBB extended the pool of HLA phenotypes through the generation of unique virtual phenotypes (VPs). A “VP database” was set up, using Microsoft Office—Access™, in order to provide NIMA-matched CBUs for potential recipients. The effectiveness of VPs’ matching was tested in 80 Greek patients. Conclusion: This methodology may contribute to the increase of the number of available CBUs for patients, in the case where there is no available CBU, or in case an additional one is needed. Through this method, the CBUs could be used faster and more effectively, rather than being cryostored for long periods of time.

## 1. Introduction

The HLA system plays a crucial role in transplantation of hematopoietic stem cells and solid organs. It displays extensive polymorphism and is characterized by the Mendelian inheritance rules, thus making donor identification efficient [1]. In the case that a graft from an individual needs to be accepted from another, HLA is used to match the patients and donors of transplants [2].

In allogeneic transplantation, the alloimune response is triggered by the activation of CD4+ T cells which induce, via cytotoxic T cells, the production of B cells and cells of the innate immune system [3]. In the case that the level of HLA matching between the donor and the recipient is high, then the T cell activation is limited, suppressing both the acute rejection response and the indirect T cell-dependent B cell responses. These responses could be the primary cause of chronic rejection and graft loss [4].

Since the first human cord blood (CB) transplantation, performed in 1988, CB banks (CBBs) have been established worldwide for the collection, processing and cryopreservation of CBUs for allogeneic hematopoietic stem cell transplantation (HSCT) [5]. Today, a global network of CBBs and transplant centers provides a common inventory. Several studies have shown that the number of hematopoietic stem cells is the most important factor for engraftment, while some degree of HLA mismatches (HLA-MM) is acceptable [6].

The advantage of using CB transplants to treat hematological malignancies rather than other sources of hematopoietic stem cells, is immediate access to the CBU with no associated risk to the donor. In addition, there could be a greater HLA disparity between donor and recipient and a decreased incidence of graft-versus-host disease [7]. A current CBU search and selection for HSCT focuses on CBUs with a high total nucleated cell (TNC) dose, from donors with the highest level of HLA matching (4/6 or better level for HLA-A, -B “low-resolution” and HLA-DRB1 “high resolution”) [7]. When using unrelated donors, HLA matching is considered to be the most important factor for CBU selection and transplantation success. To maximize graft survival, matching at the HLA-A, HLA-B, and HLA-DRBI loci is recommended (6/6 matching alleles with each loci having two alleles).

However, the immunological consequences of fetal exposure of the CBU donor to maternal cells should not be overlooked. During pregnancy, bidirectional regulation occurs in such a way that the maternal immune system tolerates the inherited paternal antigens (IPA), while the developing fetus is exposed to maternal cells expressing non-inherited maternal antigens [8]. This leads to the development of immunity and tolerance to the fetus. Tolerance towards these HLA-mismatched NIMA is thought to be mediated by the suppression of alloreactive cell expansion via regulatory T cells and/or the lysis of NIMA-specific targets via NIMA-specific cytotoxic T cells. These cytotoxic T cells can be detected in fetal blood, CB and adult peripheral blood [9,10,11,12]. In general, a CBU has one NIMA in each HLA-A, -B, and -DRB1 loci, contributing in the creation of up to 26 possible “virtual phenotypes” (VPs).

Recent research on the use of CB for unrelated stem cell transplantation has investigated the role of NIMA. When maternal HLA typing of stored CBUs is performed routinely, then patients who otherwise would receive an HLA-mismatched CBU could be transplanted with a 6/6 virtual matched CBU. Therefore, the probability of such CBUs to be selected for allogeneic transplantation is increased. Data has shown that patients who have been transplanted with CB from donors with one HLA-MM and 1 NIMA-match (1 NIMA-M) to the recipient and even from those transplanted with two HLA-MM and one NIMA-M (5/6 virtual match) to the recipient, have improved neutrophil recovery, lower transplantation-related mortality (TRM), and reduced incidence of relapse [13]. Reduced TRM and higher rates of survival were verified, independently, by an Eurocord Center for International Blood and Marrow Transplant Research study [14]. Likewise, the improved outcomes of NIMA-matched haplo-identical sibling renal and stem cell transplantations at least suggest that unrelated CBUs with three HLA-MM, where the MM are “balanced” by three NIMA-M, could also provide a higher rate of survival [15,16].

The aim of this study was to develop a system that would facilitate the identification of virtually HLA-matched CBUs for those patients who require an inherited HLA-matched CBU but who have difficulty in finding one. The identification of the NIMA haplotypes of a number of CBUs that are stored in the HCBB, yielded up to the creation of a large number of VPs, which actually “exist” only through the linkage with the “original” CBU from which they were generated. Therefore, it was possible to: (a) increase the number of HLA phenotypes of the HCBB’s registry, by accumulating the VPs in the inherited ones, and (b) create a system of grouping the CBUs of the CBB, along with their VPs, building step-by-step a database of virtual HLA phenotypes. Finally, this virtual HLA phenotype database could provide virtually-matched CBUs for patients without an available 6/6 inherited HLA-matched CB donor.

## 2. Materials and Methods

### 2.1. HLA Typing

A group of 380 CBUs of the HCBB registry were randomly selected for our study. All of them had been donated and cryostored after the mothers’ consent, during the period 2008–2012. All CB neonatal donors and their mothers were of Greek origin. The informed consent was in accordance to Helsinki declaration, conformed with National Ethical Committee and accepted by the Institutional Ethical Board.

The HLA typing of CBUs and mothers of the neonatal donors was performed at intermediate level resolution for the HLA-A, -B, and -DRB1 loci, with the PCR sequence-specific oligonucleotides (PCR-SSO) method (LIFECODES, Immucor), i.e., hybridization with synthetic oligonucleotides, after DNA amplification with PCR, using Luminex technology.

### 2.2. Identification of NIMA Haplotypes and Creation of VPs

In general, a MM in a specific HLA locus (HLA-A, -B or -DRB1) between a CBU and the mother of the neonatal donor, identifies the NIMAs, indicating a HLA that has not been inherited. In the beginning of the study, several categories of CBUs were created, depending on the number of HLA-A-B-DRB1 MM between the CBU and the mother and, hence, the number of identified NIMA that compose the NIMA haplotype.

The number of MM between the HLA phenotype of a CBU and the mother of the neonatal donor determines the number of VPs that will occur. Thus, for each CBU, all possible combinations between the NIMA and the inherited HLA have been designed, resulting in the creation of the VPs. An example of the creation of VPs after the identification of the NIMA haplotype of a specific CBU, is presented in Table 1.

### 2.3. VP Database Development

The VP database was developed using Microsoft (MS) Office Excel^TM^ and Access^TM^. In addition, diagrams regarding the created VPs and the % HLA inherited and virtual matches was performed with Graph Pad Prism v 6.01 (GraphPad Software, San Diego, CA, USA).

A “VP analysis” file of MS Office Excel^TM^ spreadsheets was developed, depending on the number of HLA MM between a CBU and the mother of the neonatal donor. For each CBU, a field was created within the relevant spreadsheet, presenting the following variables: CBU ID, the neonatal donor’s and maternal HLA-A-B-DRB1, NIMA haplotype, and the VPs that were created.

## 3. Results

### 3.1. Generation of Virtual HLA Phenotypes Registry

The maximum number of HLA-MM between a CBU and a mother is three and the same applies for the maximum number of NIMAs composing a NIMA haplotype. Thus, three basic categories of CBUs were created: (a) with one MM, (b) with two MM, and (c) with three MM between the mother and the donor. In the case that the CBU or the mother were homozygous for HLA-A, -B, and/or -DRB1, then the identified NIMAs were fewer, and hence, the CBU yielded fewer VPs. The same occurred if one had identical antigens with the other, in one or more loci.

Summarizing all categories of MM between a CBU and the mother, as well as the cases of homozygosity in any HLA phenotype of them, the number of identified NIMAs and created VPs, was calculated on a case-by-case basis, as presented in Table 2.

According to Table 2, the HLA phenotypes of 380 CBUs and the neonatal donors’ mothers were studied, as follows:

Two VPs were created, per case, after the analysis of the nine CBUs of the first category (one MM between mother and donor): resulting in 18 new VPs.

Eight VPs were created, per case, after the analysis of 79 CBUs of the second Category (two MM between mother and donor): resulting in 632 new VPs.

26 VPs were created, per case, after the analysis of 96 CBUs of the third Category (three MM between mother and donor): resulting in 2496 new VPs.

Up to 17 VPs were created after the analysis of 196 CBUs with homozygous HLA in their phenotype and/or the neonatal donor’s mother phenotype, resulting in 1827 new VPs.

**Figure 1:**
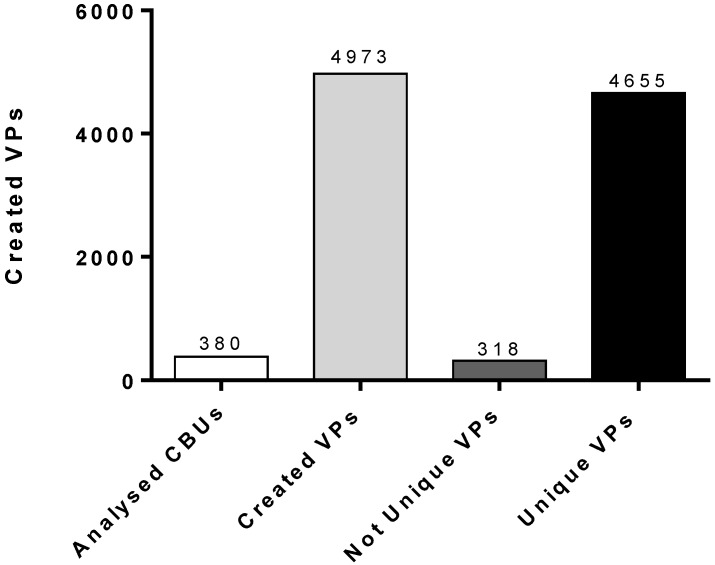
Analysed CBUs and Created VPs.

Subsequently, 4973 created VPs were examined for similarity with any of the already-existing CBUs of the HCBB. In the case that a VP was found to be identical with the HLA phenotype of a cryostored CBU, the VP was considered to be “not unique”. The result was that 318 out of the 4973 VPs were “detected” as existing HLA phenotypes of the cryostored CBUs. The other 4655 VPs were considered to be “unique”, and they can be counted in the existing pool of HLA phenotypes of the cryostored CBUs (Figure 1).

### 3.2. Creation of a Virtual Phenotype Database: A System that Provides NIMA-Matched CBUs for Patients

In order to identify the NIMA haplotype and to generate the VPs, the whole procedure of analysis and combination of CBU and mother HLA was recorded in a “VP analysis” Microsoft Excel^TM^ spreadsheet. Each VP had the following indication: ID code of the original CBU (from which it was generated) and a sequential number (Table 3). In the end, all CBUs were linked to their “virtual” HLA phenotypes, each one in the proper field of the “VP analysis” MS Office Excel^TM^ spreadsheet.

The rapid identification of CBUs that include one or more VPs that are 6/6-compatible with a recipient, was facilitated by the development of the “VP database”. This is a user-friendly search engine that is based on MS Office Access^TM^ software, which contains all the VPs that have been generated in this study. There is an obvious linkage between each VP and the original cryostored CBU, which actually exists in the registry of the HCBB. The “VP database” has been created by importing all data of the CBUs and the VPs from the “VP analysis” MS Office Excel^TM^ file. As it is presented in the next paragraph, by using the search tool of each column and by applying the required filters in each one of them, it is easy to observe only the CBUs that are NIMA-matched to a recipient with a given HLA phenotype.

### 3.3. Running the “VP Database”

**Figure 2:**
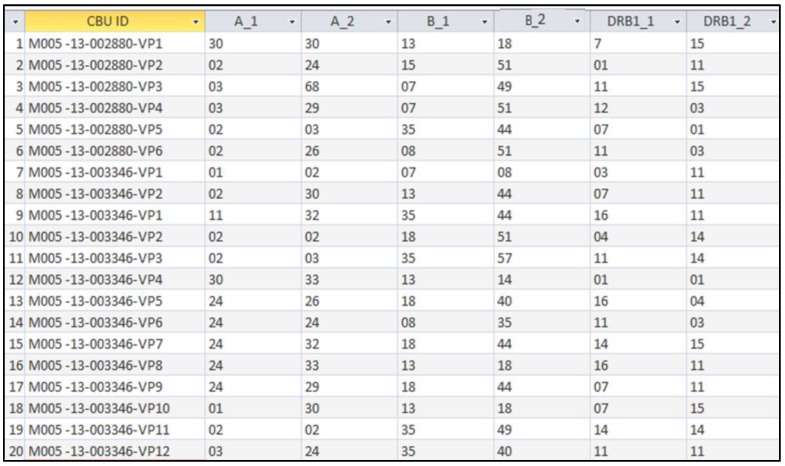
A representative screenshot of the “VP database” worksheet.

**Figure 3:**
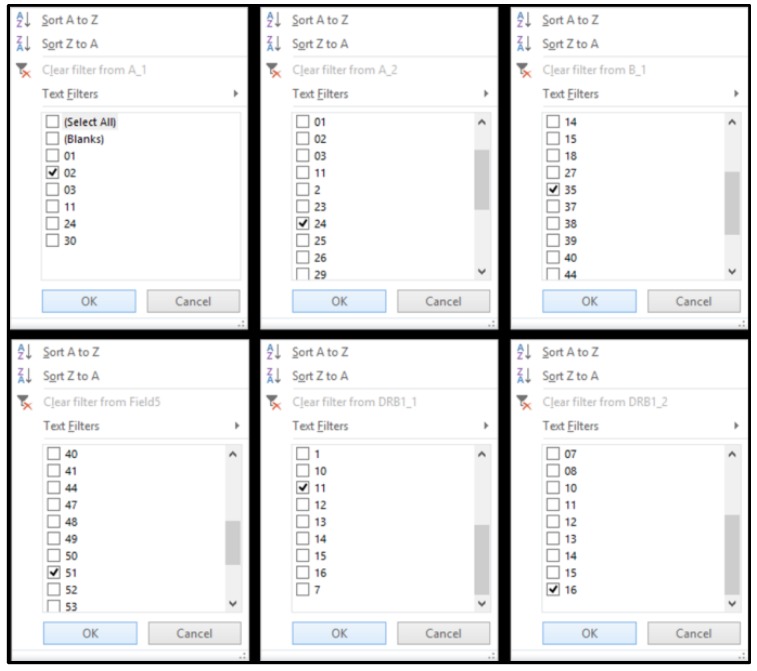
A screenshot of the filters tab that is available in each column of the “VP database” worksheet.

A hypothetical patient–recipient of a CB transplant has HLA-A, -B, and -DRB1, which are presented in Table 4. The “VP database” was capable of applying filters for each HLA column (Figure 2). In each column, the corresponding filter value was applied (Figure 3). In this example, in each column, the following filters were applied:

- value “2”: in the HLA-A_1 column,
- value “24”: in the HLA-A_2 column,
- value “35”: in the HLA-B_1 column,
- value “51”: in the HLA-B_2 column,
- value “11”: in the HLA-DRB1_1 column and
- value “16”: in the HLA-DRB1_2 column.

**Figure 4:**
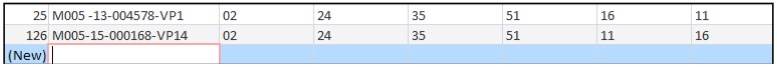
A screenshot of the “VP database”, after the application of filters based on the HLA of the patient #12345 of Table 4.

**Figure 5:**
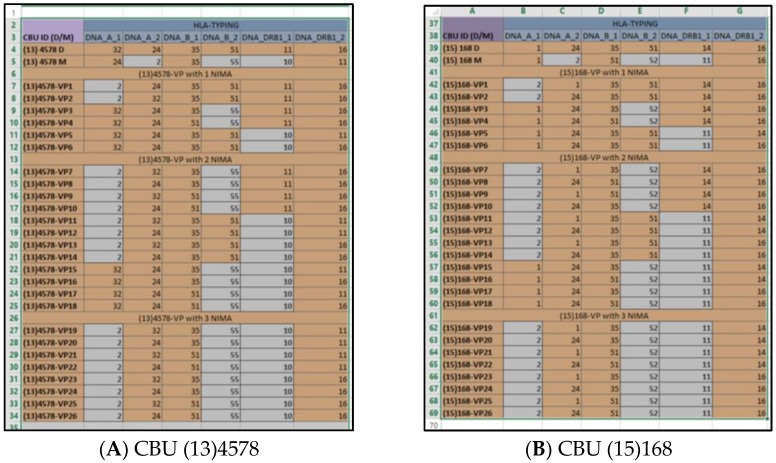
A screenshot of the field of analysis of: (**A**) CBU (13)4578 and (**B**) CBU (15)168, showing HLA-A, B and DRB1 of the CB donor, the mother, and all generated VPs.

After applying all filters, the database was "limited" in the VPs that were 6/6-matched to the HLA of the recipient. The ID code of each VP indicated the original CBU that was “virtually” 6/6-matched to the recipient (Figure 4). Thus, it appeared that the CBUs encoded as “(13)4578” and “(15)168” contained one or more NIMA that participated in a VP that was 6/6-matched to the HLA phenotype of the recipient. For more details about the original CBU that included the VP, the related field of the “VP analysis” Microsoft-Excel^TM^ could be retrieved (Figure 5).

### 3.4. The“VP Database” Can Provide Virtual Matches for Greek Patients

In 2016, 80 Greek patients were assessed for inherited HLA-matched CBUs of the HCBB, after a preliminary search request of Greek transplant centers. All patients were of European Caucasoid ethnicity.

In our study, these 80 patients were retrospectively assessed for virtually matched CBUs, via the “VP database”. The inherited-HLA-matched CBUs that had been taken into account at the time of the search request, had the following level of match: 6/6, 5/6, and 4/6. According to the Greek transplant centers’ request policies, there was no interest for CBUs with a lower match than 4/6. The virtually-NIMA-matched CBUs that were taken into account had the following level of match: virtual-6/6 (“5/6 + 1 NIMA” or “4/6 + 2 NIMA”) and virtual 5/6 (“4/6 +1 NIMA”). According to the transplant centers’ policies, the “3/6 + 1 to 3 NIMA” matches were not included in the present search, since no 3/6-inherited-HLA matches were included in the search from the beginning.

**Figure 6:**
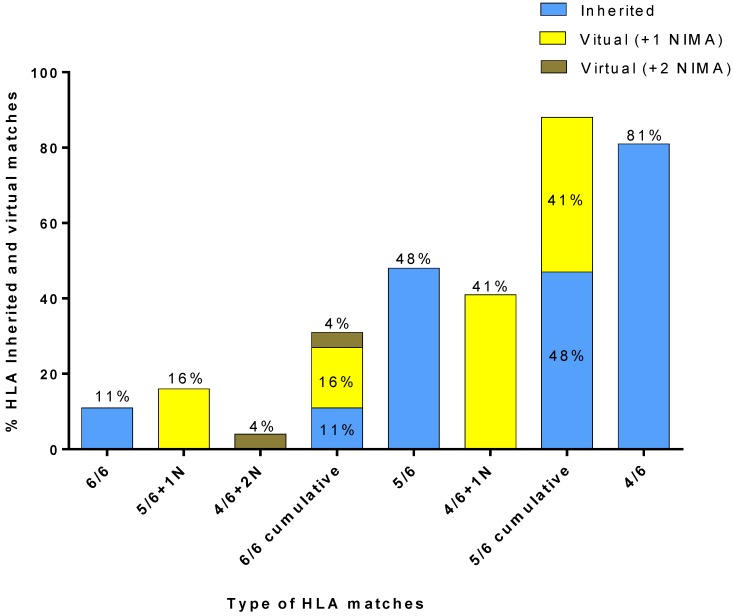
Results of matching the 80 Greek patients against the HCBB’s CBUs and the VPs of the “VP database”.

The results of matching the 80 Greek patients against the cryostored CBUs of the HCBB’s registry and the 4655 VPs of the “VP database” are presented in Figure 6. A CBU with a 6/6-inherited match was available for 11% of the 80 patients. A 5/6-matched CBU with 1 NIMA match could be identified for 16% of the patients, as well as a 4/6 CBU with two NIMA matches for 4% of the patients. Thus, out of the 80 patients, those with virtual 6/6 matches were 20%, which, when added to the patients for whom an inherited 6/6 match was found, totaled at 31%. A CBU with a 5/6-inherited match was available for 48% of the 80 patients. A 4/6-matched CBU with one NIMA match could be identified for 41% of the patients, which, when added to the patients for whom an inherited 5/6 match was found, totaled at 89%. Finally, a CBU with a 4/6-inherited match was available for 81% of the 80 patients.

## 4. Discussion

The main purpose of this study was to identify the NIMA haplotypes of the HCBB’s cryostored CBUs, in order to generate a pool of VPs. Some of these VPs would be unique and could be included in the pool of existing HLA phenotypes of the HCBB, increasing the match rate for patients with no available matched CBU.

Therefore, the HCBB proposes an alternative way of searching for NIMA-matched CBUs, by setting up the “VP database”. Such a database can be easily created by using minimum and inexpensive electronic tools. Furthermore, it does not require sophisticated software training. In our case, MS Office was chosen because it is one of the most widely used spreadsheet and database software suites in the world. The “VP database” may provide an example for CBBs on how to track VP-matched CBUs for patients, when they receive search requests from transplantation centers.

Apart from the “improvement” of the matching degree of a CBU that seems to be mismatched to a recipient, the NIMA-based strategy of selecting a CBU for transplantation, is not being applied only in cases of unavailability of a 6/6-matched transplant. A NIMA-matched CBU could be provided to a recipient, along with a 6/6-matched CBU, in order to enhance the dose of nucleated cells and hematopoietic progenitor cells, increasing the probability of a faster engraftment, and reducing the post-transplant complications. According to this study, a NIMA-matched CBU can be provided as an accompanying transplant, in order to carry out a double CB transplantation for a recipient, which is a common strategy, in order to increase the cell dose and improve the transplantation outcome [17]. With the establishment of this policy, the overall rate of CBU release from the HCBB could be increased, thus, limiting the long term storage of CBUs, which may create space issues [18].

The group of 80 Greek patients who needed a CB transplant was re-assessed to estimate the effectiveness of CBUs with identified NIMA and examined the cohort of the “VP database” in order to increase the number of virtually matched CBUs of the HCBB’s registry. Our results showed a low frequency of 6/6 and 5/6-inherited matches between the CBUs and patients. The low number of cryostored CBUs in the HCBB probably could explain the low frequency of matching Greek patients with 6/6 or even with 5/6-inherited-matched CBUs, when compared to the 4/6-matched ones. Although the 11% of these patients had an inherited HLA 6/6-matched CBU, an additional 20% of them would have found either a 5/6 CBU with one NIMA, or a 4/6 CBU with two NIMA. Likewise, although the 48% of patients had an inherited 5/6-matched CBU, an additional 41% could have found a 4/6 CBU with one NIMA. In other words, since NIMA contribute to the creation of up to 26 alternative VPs for each CBU, most patients could have been provided with one virtually-matched CBU, in the case of an unavailability of any match. At the same time, although 81% of patients found a 4/6 inherited match and only 48% of them found a 5/6 inherited match, the cumulative 5/6 matched (inherited and virtual) group of patients (89%) ends up exceeding the 4/6-inherited-matched group. This may suggest that NIMA identification can “upgrade” the matching of CBUs with patients and improve patients’ access to a virtually 6/6 or 5/6-matched hematopoietic stem cell transplants. Thus, it is a huge benefit for Greek patients to find a virtual-6/6 or 5/6 match, since the 6/6 and 5/6-match rate increases for both groups from 11% to 31% for 6/6 matches, and from 48% to 89%, if the NIMA haplotypes are to be taken into consideration.

The findings above are in accordance with previous works that generated VPs by using specific algorithms, in order to estimate their effectiveness in patient groups [19,20,21]. However, such algorithms demand sophisticated software tools and greater programming knowledge in order to accomplish the same goal. Van der Zanden et al. [19] concluded that “5/6 + 1 NIMA”-matched CBUs were identified for 9.6% of the patients and “4/6 + 2 NIMA”-matched ones were identified for 7.9% of them. In our case, “5/6 + 1 NIMA”-matched CBUs were identified for 16% of the patients, and “4/6 + 2 NIMA”-matched ones were identified for 4% of them. However, the cumulative number of patients with a 6/6-matched CBU is similar in both studies: 32% in Van der Zanden’s study, and 31% in our study [19]. Powley et al. [20] and Kwok et al. [21] concluded this in different percentages of patients, but in both studies, an increase in the overall match rates was observed. The main reason for why there are minor differences in some virtual-matching subgroups’ results may be due to the limited number of CBUs in the registry of the HCBB, which influences the effectiveness of inheritance or virtual-matching between the group of patients and the CBUs.

The probability and efficiency of providing a well-matched unrelated CB donor will be significantly improved in the case that high resolution typing is available for the patient prior to the search being done. The HCBB has already begun to perform HLA typing of donors and mothers using next generation sequencing (NGS) methodology, as it provides both high-throughput and high-resolution capabilities. In the future, the HCBB plans to integrate HLA sequencing data of the CBUs in the “VP database”. It may appear that maternal HLA typing in order to identify the NIMA CBUs, would increase the HLA typing cost [22], although this cost can be reduced with the use of NGS methodology [23].

The cell dose of the CBUs that were selected for the NIMA analysis method was not taken into account. The reason is that we plan to process all the cryostored CBUs of the HCBB, no matter whether there is a high or low number of cells. For instance, in the past, we stored CBUs containing <70 × 10^7^ cells, but since 2013, we store CBUs containing >100 × 10^7^ cells. Through this way, all kind of alternative cell doses of CBUs could be identified as virtual matches for patients. Additionally, some patients with an inherited-HLA-matched CBU may need an additional one, in order to carry out a double CBU transplantation. In that case, a virtually-matched CBU could be a good choice for the transplant centers, independently from the cell dose. At the time being, CBUs with <100 × 10^7^ cells are not cryostored, despite the fact that such CBUs could “include” useful NIMA and, hence, unique VPs. Unfortunately, the processing, cryostoring, testing, and HLA typing of those CBUs, would elevate the operating expenses of the HCBB, and so the NIMA methodology focuses on CBUs with higher cell doses.

The primary limitation of this study is the small number of CBUs that are cryostored in the HCBB, in comparison to other CBBs. This affects the ability to identify available inherited or virtual matches for patients in general. Concerning the estimation of VP matching effectiveness in Greek patients, a larger group of patients would probably represent better the Greek population. In comparison to other studies that include high resolution typing of HLA-DRB1 [21,22,23], we did not include allele-level typing for all three HLA loci. Another limitation is that, due to the Greek Transplant Centers’ policies, we were not able to re-assess the 3/6-inherited HLA-matches in our retrospective study. If we had included them in the patient-matched CBU reports from the beginning, we would be able to identify which ones could provide an “upgraded” match for patients, due to their compatible NIMA. However, this is a very good idea for our future plans, to also extend the NIMA methodology in the 3/6 matches.

Further work will focus on the generation of VPs for the whole inventory of the HCBB, whereas the effectiveness of VP-matching should be estimated on a larger number of patients. Βetter assessment of the sample’s special parameters, such as the ethnicity, the weight, or the age of the patients, and the cellular content of the CBUs, is necessary, because they are critical for CBU selection for HSCT. Furthermore, HLA-C and allele-level resolution should be included in the future. An analysis of the HLA profile of the CB donors and mothers (and therefore also NIMA) could also provide important information regarding the HLA frequencies in the donor pool available to patients.

## 5. Conclusions

The NIMA identification and generation of VPs keeps evolving in order to cover up the CBUs that have been stored in the past, as well as the newly cryostored ones. The VPs that were created from the identified NIMA haplotypes in this study, could be used in the future for the estimation of the probability of finding a suitable donor for stem cell transplantation, for any Greek patient that is relying on the frequencies of HLA allelles and allele groups of the Greek population. The frequencies of VPs in the HCBB could be used for a comparison with HLA allele frequencies in ethnic minorities of the Greek population. The NIMA approach should be considered in the future from local CBBs and registries all around the world. Through this way, the chance of higher HLA matches will improve the transplantation outcome, and this may increase the utilization of CBUs.

## Figures and Tables

**Table 1:** Virtual human leucocyte antigen (HLA) phenotypes, created with the contribution of non-inherited maternal antigens (NIMA). Bold text indicates understanding how NIMA haplotype yields to 26 different HLA phenotypes.

Cord Blood Unit (CBU)	A1, A3; B7, B44; DRB1*4, DRB1*16	NIMA Haplotype: A2, B8, DRB1*11
Mother of CB Donor	A1, A2; B7, B8; DRB1*4, DRB1*11	
Combining the NIMA-A2;B8;DRB1*11 with the CB phenotype, the following virtual phenotypes (VPs) may be generated:		
One substitution		
VP1: A1, **A2**; B7, B44, DRB1*4, DRB1*16	VP3: A1, A3; **B8**, B44; DRB1*4, DRB1*16	VP5: A1, A3; B7, B44; DRB1*4, DRB1*11
VP2: **A2**, A3; B7, B44, DRB1*4, DRB1*16	VP4: A1, A3; B7, **B8**; DRB1*4, DRB1*16	VP6: A1, A3; B7, B44; DRB1*11, DRB1*16
Two substitutions		
VP7: A1, **A2**; **B8**, B44, DRB1*4, DRB1*16	VP11: **A2**, A3; **B8**, B44, DRB1*4, DRB1*16	VP15: A1, A3; **B8**, B44; DRB1*4, DRB1*11
VP8: A1, **A2**; B7, **B8**, DRB1*4, DRB1*16	VP12: **A2**, A3; B7, **B8**, DRB1*4, DRB1*16	VP16: A1, A3; B7, **B8**; DRB1*4, DRB1*11
VP9: A1, A3; **B8**, B44; DRB1*11, DRB1*16	VP13: A1, **A2**; B7, B44, DRB1*4, DRB1*11	VP17: **A2**, A3; B7, B44, DRB1*4, DRB1*11
VP10: A1, A3; B7, **B8**; DRB1*11, DRB1*16	VP14: A1, **A2**; B7, B44, DRB1*11, DRB1*16	VP18: **A2**, A3; B7, B44, DRB1*11, DRB1*16
Three substitutions		
VP19: A1, **A2**; **B8**, B44, DRB1*4, DRB1*11	VP22: A1, **A2**; **B8**, B44, DRB1*11, DRB1*16	VP25: **A2**, A3; **B8**, B44, DRB1*4, DRB1*11
VP20: A1, **A2**; B7, **B8**, DRB1*4, DRB1*11	VP23: A1, **A2**; B7, **B8**, DRB1*11, DRB1*16	VP26: **A2**, A3; B7, **B8**, DRB1*4, DRB1*11
VP21: **A2**, A3; **B8**, B44, DRB1*11, DRB1*16	VP24: **A2**, A3; B7, **B8**, DRB1*11, DRB1*16	

**Table 2:** Summary of the number of the VPs that was created for each category of CBUs, depending on the mismatch (MM) between the CBU and the neonatal donor’s mother.

Category of CBUs	MM between Donor and Mother	Loci with Homozygous HLA		Analysed CBUs	Created VPs	New, Unique VPs in the HCBB
		CBU	Neonatal Donor’s Mother			
First	1	0	0	9	18	
Second	2	0	0	79	632	
Third	3	0	0	96	2496	
Homozygous HLA	1 to 3	1 to 3	1 to 3	196	1827	
Sum	-	-	-	380	4973	4655

**Table 3:** A representative screenshot of the “VP analysis” Microsoft (MS) Office Excel^TM^ spreadsheet.

Example of Linkage between a CBU and the Created VPs	HLA Τyping					
CBU ID	HLA-A_1	HLA-A_2	HLA-B_1	HLA-B_2	HLA-DRB1_1	HLA-DRB1_2
(14)3530 D (CBU Donor)	24	24	35	51	11	16
(14)3530 M (Mother)	2	24	35	51	13	16
ΝΙΜA Haplotype	2		-		13	
(14)3530-VP 1	2	24	35	51	11	16
(14)3530-VP 2	24	24	35	51	13	16
(14)3530-VP 3	24	24	35	51	11	13
(14)3530-VP 4	2	24	35	51	13	16
(14)3530-VP 5	2	24	35	51	11	13

**Table 4:** An example of HLA-A, B, and DRB1 of a hypothetical patient.

Example	HLA-A_1	HLA-A_2	HLA-B_1	HLA-B_2	HLA-DRB1_1	HLA-DRB1_2
Patient-Recipient #12345	2	24	35	51	11	16
